# Supplementary material for: Biosynthetic Pathways of Tryptophan Metabolites in Saccharomyces cerevisiae Strain: Insights and Implications
Source: Int J Mol Sci. 2024 Apr 26;25(9):4747. doi: 10.3390/ijms25094747 (PMC11083699; doi:10.3390/ijms25094747)
Supplement: Supplementary file 1 [file ijms-25-04747-s001.zip › ijms-2918705-supplementary.pdf]

Table S1. Concentrations of tryptophan metabolites produced by the yeast grown in YPD media (Run 1).

Metabolites	Concentrations (mg L^{-1})													
	0 h		4 h		8 h		12 h		16 h		20 h		24 h	
	Mean	Std	Mean	Std	Mean	Std	Mean	Std	Mean	Std	Mean	Std	Mean	Std
3-hydroxykynurenine	ND	-	ND	-	ND	-	ND	-	ND	-	ND	-	ND	-
Serotonin	ND	-	ND	-	ND	-	ND	-	ND	-	ND	-	ND	-
5-hydroxytryptophan	0.07	0.00985	0.07	0.0154	0.07	0.00639	0.09	0.0143	0.09	0.0144	0.09	0.00583	0.08	0.00261
Kynurenine	0.221	0.0198	0.275	0.0316	0.345	0.0453	0.433	0.0762	0.456	0.0707	0.438	0.0812	0.541	0.0545
3-hydroxyanthranilic acid	ND	-	0.0152	0.00249	0.0286	0.00489	0.0644	0.00635	0.0501	0.00967	0.0453	0.0123	0.0780	0.0180
Tryptamine	0.0599	0.00229	0.0552	0.00541	0.0558	0.00713	0.0608	0.00327	0.0557	0.00185	0.0571	0.00360	0.0535	0.00465
5-hydroxyindole acetic acid	ND	-	ND	-	ND	-	ND	-	ND	-	ND	-	ND	-
N-Acetylserotonin	ND	-	ND	-	ND	-	ND	-	ND	-	ND	-	ND	-
Xanthurenic acid	ND	-	ND	-	ND	-								
Indole-3-acetamide	ND	-	ND	-	ND	-	ND	-	ND	-	ND	-	ND	-
Kynurenic acid	0.00827	0.000252	0.00843	0.000757	0.0109	0.000872	0.0196	0.00240	0.0185	0.000603	0.0192	0.00296	0.0288	0.00412
DL-Indole-3-lactic acid	3.00	0.0679	2.87	0.109	2.65	0.115	2.67	0.0584	2.58	0.00680	2.62	0.0731	2.42	0.121
Indole-3-carboxaldehyde	0.0370	0.000351	0.0383	0.000808	0.0416	0.00323	0.0410	0.000709	0.0412	0.00229	0.0401	0.00296	0.0384	0.00121
Indole-3-acetic acid	0.0525	0.00257	0.0519	0.00681	0.0513	0.00479	0.0587	0.00199	0.0561	0.00220	0.0572	0.00262	0.0592	0.00486
Tryptophol	0.006	0.00149	0.0103	0.000757	0.0181	0.000954	0.0353	0.0121	0.0357	0.00166	0.0379	0.00948	0.0892	0.0299
Melatonin	ND	-	ND	-	ND	-	ND	-	ND	-	ND	-	ND	-

Table S2. Concentrations of tryptophan metabolites produced by the yeast grown in YPD media with 50% of peptone replaced by tryptophan (Run 2).

Metabolites	Concentrations (mg L^{-1})													
	0 h		4 h		8 h		12 h		16 h		20 h		24 h	
	Mean	Std	Mean	Std	Mean	Std	Mean	Std	Mean	Std	Mean	Std	Mean	Std
3-hydroxykynurenine	ND	-												
Serotonin	ND	-	ND	-	ND	-	ND	-	ND	-	ND	-	ND	-
5-hydroxytryptophan	4.09	0.400	4.40	0.857	5.14	0.726	4.82	1.45	4.87	0.896	5.26	0.460	6.17	0.676
Kynurenine	3.85	0.0712	4.87	0.330	5.95	0.838	6.78	0.361	6.35	1.31	8.90	1.63	8.48	0.110
3-hydroxyanthranilic acid	0.00833	0.000808	0.0358	0.000917	0.0624	0.00761	0.0882	0.00712	0.0993	0.0189	0.115	0.0202	0.101	0.00535
Tryptamine	2.33	0.150	2.41	0.111	2.36	0.161	2.38	0.062	2.11	0.183	2.44	0.0413	2.42	0.0960
5-hydroxyindole acetic acid	ND	-	ND	-	ND	-	ND	-	ND	-	ND	-	ND	-
N-Acetylserotonin	ND	-	ND	-	ND	-	ND	-	ND	-	ND	-	ND	-
Xanthurenic acid	ND	-	0.0416	0.00705	0.104	0.00691	0.178	0.0696	0.289	0.0595	0.354	0.0910	0.369	0.138
Indole-3-acetamide	0.00217	0.000351	0.00190	0.000656	0.0005	0.000866	0.00280	0.000436	0.00512	0.00124	0.00410	0.000781	0.00333	0.00110
Kynurenic acid	0.0175	0.00205	0.0322	0.00193	0.0612	0.00322	0.0782	0.0142	0.115	0.0204	0.149	0.0277	0.150	0.0317
DL-Indole-3-lactic acid	43.0	0.858	42.5	0.167	41.1	1.74	41.1	0.581	39.3	1.05	40.3	0.348	41.1	0.829
Indole-3-carboxaldehyde	0.323	0.0110	0.449	0.00564	0.650	0.0438	0.711	0.0365	0.598	0.0423	0.688	0.0476	0.664	0.0232
Indole-3-acetic acid	0.108	0.00567	0.148	0.0192	0.219	0.0187	0.249	0.00815	0.247	0.0145	0.3096	0.0335	0.2938	0.00870
Tryptophol	0.2980	0.00284	0.7496	0.0157	1.96	0.0510	3.48	0.389	3.62	0.112	3.77	0.0406	3.95	0.283
Melatonin	ND	-	ND	-	ND	-	ND	-	ND	-	ND	-	ND	-

Table S3. Concentrations of tryptophan metabolites produced by the yeast grown in YPD media with complete replacement of peptone by tryptophan (Run 3).

Metabolites	Concentrations (mg L^{-1})													
	0 h		4 h		8 h		12 h		16 h		20 h		24 h	
	Mean	Std	Mean	Std	Mean	Std	Mean	Std	Mean	Std	Mean	Std	Mean	Std
3-hydroxykynurenine	ND	-	ND	-	ND	-	ND	-	ND	-	ND	-	ND	-
Serotonin	0.0585	0.000917	0.0627	0.00373	0.0643	0.00426	0.0604	0.00432	0.0596	0.00235	0.0637	0.00181	0.0612	0.00554
5-hydroxytryptophan	45.5	6.82	42.3	2.47	51.5	4.94	46.1	0.91	46.8	2.62	47.3	2.01	50.0	1.95
Kynurenine	3.82	0.590	3.92	0.517	4.92	0.628	5.15	0.338	5.68	0.564	6.23	0.943	6.62	0.850
3-hydroxyanthranilic acid	2.88	1.84	9.74	2.72	22.7	6.98	26.8	1.57	41.2	6.23	38.6	2.34	41.4	4.48
Tryptamine	16.1	2.44	15.8	0.554	15.0	1.02	12.5	0.77	12.5	0.881	12.8	0.244	13.2	0.231
5-hydroxyindole acetic acid	ND	-	ND	-	ND	-	ND	-	ND	-	ND	-	ND	-
N-Acetylserotonin	ND	-	ND	-	ND	-	ND	-	ND	-	ND	-	ND	-
Xanthurenic acid	0.0345	0.00410	0.0649	0.00637	0.186	0.0517	0.274	0.0218	0.551	0.131	0.509	0.107	0.513	0.0827
Indole-3-acetamide	ND	-	ND	-	ND	-	ND	-	ND	-	ND	-	ND	-
Kynurenic acid	0.0433	0.00257	0.0636	0.00271	0.118	0.0215	0.179	0.0128	0.314	0.0566	0.295	0.0409	0.287	0.0286
DL-Indole-3-lactic acid	8.56	0.665	7.79	0.242	7.07	0.161	6.90	0.165	7.02	0.0664	7.21	0.167	7.06	0.150
Indole-3-carboxaldehyde	0.589	0.0683	0.737	0.0860	0.970	0.0955	0.967	0.0640	1.02	0.0697	1.07	0.111	0.929	0.174
Indole-3-acetic acid	0.229	0.0299	0.287	0.0639	0.438	0.0291	0.503	0.0434	0.517	0.0131	0.554	0.0337	0.564	0.0301
Tryptophol	1.46	0.167	4.40	0.360	11.7	2.58	19.1	0.586	19.2	0.493	19.2	1.03	19.1	0.279
Melatonin	ND	-	ND	-	ND	-	ND	-	ND	-	ND	-	ND	-

Table S4. Concentrations of tryptophan metabolites produced by the yeast grown in YPD media with complete replacement of peptone by tryptophan and with the addition of 25 mM HEPES buffer (Run 4).

Metabolites	Concentrations (mg L^{-1})													
	0 h		4 h		8 h		12 h		16 h		20 h		24 h	
	Mean	Std	Mean	Std	Mean	Std	Mean	Std	Mean	Std	Mean	Std	Mean	Std
3-hydroxykynurenine	ND	-	ND	-	ND	-	ND	-	ND	-	ND	-	ND	-
Serotonin	0.0632	0.00642	0.0613	0.00159	0.0598	0.0062	0.0602	0.00127	0.0633	0.00119	0.0644	0.00272	0.0650	0.00211
5-hydroxytryptophan	58.9	16.0	48.3	3.98	51.2	5.25	51.20	3.42	57.0	2.70	56.0	4.29	56.7	6.92
Kynurenine	4.76	0.434	5.38	0.267	5.54	0.321	5.15	0.446	5.84	1.19	6.38	1.92	7.38	1.30
3-hydroxyanthranilic acid	1.67	0.280	22.1	1.30	32.6	3.75	33.3	5.40	48.8	12.7	113	131	119	134
Tryptamine	18.0	1.42	18.1	1.04	14.5	0.999	11.8	0.392	12.8	1.88	13.4	0.852	14.1	1.38
5-hydroxyindole acetic acid	ND	-	ND	-	ND	-	ND	-	ND	-	ND	-	ND	-
N-Acetylserotonin	ND	-	ND	-	ND	-	ND	-	ND	-	ND	-	ND	-
Xanthurenic acid	0.0449	0.0163	0.0677	0.00279	0.143	0.0122	0.216	0.0578	0.277	0.107	0.541	0.580	0.597	0.651
Indole-3-acetamide	ND	-	ND	-	ND	-	ND	-	ND	-	ND	-	ND	-
Kynurenic acid	0.0423	0.00302	0.0645	0.00385	0.112	0.00887	0.157	0.0349	0.215	0.0557	0.265	0.176	0.272	0.1560
DL-Indole-3-lactic acid	7.95	0.226	7.98	0.0849	7.17	0.272	6.74	0.175	6.98	0.4641	6.99	0.182	7.11	0.340
Indole-3-carboxaldehyde	0.5801	0.0235	0.783	0.0310	1.21	0.374	1.0	0.0950	1.12	0.257	1.16	0.278	1.10	0.237
Indole-3-acetic acid	0.589	0.0458	0.619	0.0126	0.688	0.0375	0.688	0.044	0.718	0.0789	0.820	0.0789	0.780	0.0422
Tryptophol	1.10	0.0464	3.02	0.0178	13.2	1.16	17.0	0.874	20.6	1.16	20.8	2.02	22.6	1.87
Melatonin	ND	-	ND	-	ND	-	ND	-	ND	-	ND	-	ND	-

Table S5. Concentrations of tryptophan metabolites produced by the yeast grown in YPD media with complete replacement of peptone by tryptophan and with the addition of 100 mM HEPES buffer (Run 5).

Metabolites	Concentrations (mg L^{-1})													
	0 h		4 h		8 h		12 h		16 h		20 h		24 h	
	Mean	Std	Mean	Std	Mean	Std	Mean	Std	Mean	Std	Mean	Std	Mean	Std
3-hydroxykynurenine	ND	-	ND	-	ND	-	ND	-	ND	-	ND	-	ND	-
Serotonin	ND	-	ND	-	ND	-	ND	-	ND	-	ND	-	ND	-
5-hydroxytryptophan	3.40	0.238	2.79	0.243	2.87	0.183	3.04	0.199	3.08	0.0808	2.92	0.0473	3.24	0.0935
Kynurenine	0.63	0.0504	0.75	0.05698	0.92	0.0709	1.06	0.111	1.26	0.223	1.17	0.160	1.43	0.231
3-hydroxyanthranilic acid	0.01	0.00095	0.02	0.000833	0.04	0.00280	0.05	0.00619	0.08	0.03671	0.10	0.0230	0.10	0.0178
Tryptamine	3.24	0.254	3.12	0.241	2.92	0.126	2.87	0.220	2.88	0.0454	2.80	0.0950	2.90	0.0805
5-hydroxyindole acetic acid	ND	-	ND	-	ND	-	ND	-	ND	-	ND	-	ND	-
N-Acetylserotonin	ND	-	ND	-	ND	-	ND	-	ND	-	ND	-	ND	-
Xanthurenic acid	ND	-	4.42	0.686	11.4	1.36	18.1	2.33	32.1	13.5	60.0	19.5	48.4	16.9
Indole-3-acetamide	0.10	0.00420	0.10	0.00471	0.10	0.00796	0.11	0.0117	0.10	0.000929	0.10	0.00165	0.11	0.00871
Kynurenic acid	0.04	0.00180	0.05	0.00361	0.07	0.00465	0.08	0.00577	0.13	0.0290	0.17	0.0295	0.17	0.0258
DL-Indole-3-lactic acid	3586	535	3304	249	3971	425	2826	67.1	3858	440	3142	583	3440	494
Indole-3-carboxaldehyde	0.28	0.00338	0.38	0.0314	0.57	0.0387	0.73	0.101	0.84	0.061	0.75	0.0779	0.83	0.0977
Indole-3-acetic acid	1.22	0.170	1.26	0.0616	1.49	0.0809	1.56	0.151	1.56	0.0290	1.59	0.100	1.72	0.0906
Tryptophol	0.34	0.00703	0.840	0.0327	3.00	0.469	7.09	0.201	7.02	0.497	7.19	0.194	7.03	0.260
Melatonin	ND	-	ND	-	ND	-	ND	-	ND	-	ND	-	ND	-

Table S6. Concentrations of tryptophan metabolites produced by the yeast grown in YPD media with complete replacement of peptone by tryptophan and with the addition of 0.1% Tween 20 (Run 6).

Metabolites	Concentrations (mg L^{-1})													
	0 h		4 h		8 h		12 h		16 h		20 h		24 h	
	Mean	Std	Mean	Std	Mean	Std	Mean	Std	Mean	Std	Mean	Std	Mean	Std
3-hydroxykynurenine	ND	-	ND	-	ND	-	ND	-	ND	-	ND	-	ND	-
Serotonin	ND	-	ND	-	ND	-	ND	-	ND	-	ND	-	ND	-
5-hydroxytryptophan	2.63	0.241	2.44	0.0915	2.67	0.290	2.31	0.196	2.73	0.326	2.55	0.300	2.78	0.1178
Kynurenine	0.86	0.105	1.01	0.0126	1.12	0.126	1.04	0.0218	1.33	0.101	1.53	0.257	1.69	0.0500
3-hydroxyanthranilic acid	0.01	0.000436	0.02	0.00422	0.04	0.00768	0.05	0.00215	0.07	0.0185	0.100	0.0137	0.110	0.0111
Tryptamine	2.99	0.254	2.84	0.0883	2.78	0.345	2.59	0.130	2.89	0.0630	2.77	0.167	2.77	0.182
5-hydroxyindole acetic acid	ND	-	ND	-	ND	-	ND	-	ND	-	ND	-	ND	-
N-Acetylserotonin	ND	-	ND	-	ND	-	ND	-	ND	-	ND	-	ND	-
Xanthurenic acid	ND	-	10.1	2.19	30.4	5.43	48.3	11.3	80.0	26.8	127	21.3	125	15.0
Indole-3-acetamide	0.110	0.00517	0.11	0.00806	0.110	0.00391	0.100	0.00791	0.100	0.0103	0.110	0.00380	0.110	0.00892
Kynurenic acid	0.0400	0.00163	0.0600	0.00531	0.110	0.00771	0.180	0.0315	0.250	0.0712	0.380	0.0600	0.380	0.0427
DL-Indole-3-lactic acid	2870	457	2831	177	2904	568	2411	160	2968	222	2617	251	2501	154
Indole-3-carboxaldehyde	0.290	0.00976	0.390	0.00949	0.570	0.0510	0.520	0.0323	0.620	0.0198	0.660	0.126	0.680	0.0243
Indole-3-acetic acid	0.360	0.0226	0.470	0.0362	0.680	0.151	0.790	0.0481	0.940	0.138	1.01	0.126	0.930	0.147
Tryptophol	0.380	0.00859	0.900	0.0797	3.41	0.742	3.92	0.931	4.09	0.994	3.28	0.192	3.10	0.0466
Melatonin	ND	-	ND	-	ND	-	ND	-	ND	-	ND	-	ND	-

Table S7. Concentrations of tryptophan metabolites produced by the yeast grown in YPD media with complete replacement of peptone by tryptophan and with the addition of 0.1% Tween 20 and 25 mM HEPES buffer (Run 7).

Metabolites	Concentrations (mg L^{-1})													
	0 h		4 h		8 h		12 h		16 h		20 h		24 h	
	Mean	Std	Mean	Std	Mean	Std	Mean	Std	Mean	Std	Mean	Std	Mean	Std
3-hydroxykynurenine	ND	-	ND	-	ND	-	ND	-	ND	-	ND	-	ND	-
Serotonin	ND	-	ND	-	ND	-	ND	-	ND	-	ND	-	ND	-
5-hydroxytryptophan	3.33	0.249	2.84	0.112	3.04	0.187	3.26	0.205	3.12	0.202	3.33	0.0526	3.39	0.182
Kynurenine	1.00	0.0462	0.94	0.0496	1.17	0.0598	1.45	0.0124	1.39	0.198	1.36	0.125	1.86	0.625
3-hydroxyanthranilic acid	0.01	0.000723	0.02	0.00156	0.06	0.0718	0.04	0.00596	0.04	0.0296	0.07	0.0145	0.04	0.00191
Tryptamine	2.82	0.183	2.48	0.0859	2.63	0.182	2.51	0.238	2.39	0.0398	2.47	0.0803	2.47	0.180
5-hydroxyindole acetic acid	ND	-	ND	-	ND	-	ND	-	ND	-	ND	-	ND	-
N-Acetylserotonin	ND	-	ND	-	ND	-	ND	-	ND	-	ND	-	ND	-
Xanthurenic acid	0.46	0.0910	1.07	0.176	5.95	0.229	21.61	6.72	26.0	29.6	40.6	15.5	27.3	9.11
Indole-3-acetamide	ND	-	ND	-	ND	-	ND	-	ND	-	ND	-	ND	-
Kynurenic acid	0.03	0.00212	0.03	0.00155	0.04	0.00398	0.09	0.0266	0.11	0.0839	0.15	0.0300	0.12	0.0241
DL-Indole-3-lactic acid	3231	207	2448	326	2997	718	3097	500	2488	651	2906	274	2922	193
Indole-3-carboxaldehyde	0.31	0.0310	0.35	0.0208	0.50	0.0374	0.71	0.0638	0.64	0.110	0.61	0.0180	0.70	0.0275
Indole-3-acetic acid	0.31	0.00732	0.31	0.0271	0.37	0.0234	0.46	0.0372	0.45	0.0729	0.50	0.0255	0.54	0.0188
Tryptophol	0.11	0.00934	0.37	0.0231	1.89	0.0713	5.87	0.424	6.26	1.81	7.22	0.0454	8.03	0.244
Melatonin	ND	-	ND	-	ND	-	ND	-	ND	-	ND	-	ND	-

Table S8. Concentrations of tryptophan metabolites produced by the yeast grown in YPD media with complete replacement of peptone by tryptophan and with the addition of 0.1% Tween 20 and 100 mM HEPES buffer (Run 8).

Metabolites	Concentrations (mg L^{-1})													
	0 h		4 h		8 h		12 h		16 h		20 h		24 h	
	Mean	Std	Mean	Std	Mean	Std	Mean	Std	Mean	Std	Mean	Std	Mean	Std
3-hydroxykynurenine	ND	-	ND	-	ND	-	ND	-	ND	-	ND	-	ND	-
Serotonin	ND	-	ND	-	ND	-	ND	-	ND	-	ND	-	ND	-
5-hydroxytryptophan	4.02	0.411	3.59	0.0833	3.73	0.0915	3.87	0.121	3.85	0.0624	3.82	0.179	4.61	1.46
Kynurenine	1.15	0.131	1.19	0.102	1.28	0.0696	1.36	0.0856	1.55	0.0997	1.58	0.174	2.04	0.654
3-hydroxyanthranilic acid	ND	-	ND	-	ND	-	ND	-	ND	-	ND	-	ND	-
Tryptamine	4.41	0.452	3.79	0.116	3.91	0.0906	3.88	0.128	3.90	0.132	3.94	0.0594	4.71	1.44
5-hydroxyindole acetic acid	ND	-	ND	-	ND	-	ND	-	ND	-	ND	-	ND	-
N-Acetylserotonin	ND	-	ND	-	ND	-	ND	-	ND	-	ND	-	ND	-
Xanthurenic acid	ND	-	ND	-	ND	-	ND	-	ND	-	ND	-	ND	-
Indole-3-acetamide	ND	-	ND	-	ND	-	ND	-	ND	-	ND	-	ND	-
Kynurenic acid	0.02	0.000578	0.01	0.00620	0.02	0.00460	0.02	0.00488	0.01	0.00633	0.01	0.00900	0.02	0.00245
DL-Indole-3-lactic acid	3285	654	4334	1994	3450	112	3401	69.1	3390	141	3820	427	4177	1200
Indole-3-carboxaldehyde	0.26	0.0370	0.280	0.00434	0.310	0.00647	0.310	0.00429	0.330	0.00935	0.340	0.00552	0.380	0.0297
Indole-3-acetic acid	1.59	0.107	1.31	0.0700	1.37	0.0668	1.37	0.0245	1.47	0.0966	1.38	0.0601	1.70	0.659
Tryptophol	ND	-	ND	-	ND	-	0.0200	0.00686	0.0500	0.0316	0.140	0.0192	0.34	0.181
Melatonin	ND	-	ND	-	ND	-	ND	-	ND	-	ND	-	ND	-

Table S9. Concentrations of tryptophan metabolites produced by the yeast grown in YPD media with complete replacement of peptone by tryptophan and with the addition of 0.2% Tween 20 (Run 9).

Metabolites	Concentrations (mg L^{-1})													
	0 h		4 h		8 h		12 h		16 h		20 h		24 h	
	Mean	Std	Mean	Std	Mean	Std	Mean	Std	Mean	Std	Mean	Std	Mean	Std
3-hydroxykynurenine	ND	-	ND	-	0.0100	0.00452	0.0100	0.00316	0.0400	0.0291	0.0400	0.00513	0.0400	0.0216
Serotonin	ND	-	ND	-	ND	-	ND	-	ND	-	ND	-	ND	-
5-hydroxytryptophan	2.52	0.174	2.40	0.147	2.62	0.0429	2.43	0.332	2.71	0.0306	2.48	0.0736	2.49	0.334
Kynurenine	0.77	0.0506	0.840	0.0735	1.05	0.0214	1.02	0.0585	1.36	0.102	1.34	0.0468	1.50	0.0753
3-hydroxyanthranilic acid	ND	-	0.0200	0.00231	0.0400	0.00722	0.0700	0.0145	0.100	0.0832	0.150	0.0435	0.180	0.0792
Tryptamine	3.14	0.125	2.83	0.238	2.85	0.0754	2.71	0.215	2.91	0.100	2.76	0.157	2.75	0.268
5-hydroxyindole acetic acid	ND	-	ND	-	ND	-	ND	-	ND	-	ND	-	ND	-
N-Acetylserotonin	ND	-	ND	-	ND	-	ND	-	ND	-	ND	-	ND	-
Xanthurenic acid	4.57	0.258	16.0	1.20	36.4	3.01	58.3	13.7	86.9	65.2	107	36.9	119	71.7
Indole-3-acetamide	ND	-	ND	-	ND	-	ND	-	ND	-	ND	-	ND	-
Kynurenic acid	0.0400	0.000867	0.09	0.00686	0.170	0.0135	0.300	0.0610	0.380	0.277	0.520	0.137	0.560	0.228
DL-Indole-3-lactic acid	1931	29.3	2258	184	2300	173	2640	646	2486	335	2402	465	2403	202
Indole-3-carboxaldehyde	0.230	0.0109	0.290	0.0131	0.450	0.0253	0.400	0.0311	0.470	0.0257	0.410	0.0360	0.430	0.0680
Indole-3-acetic acid	0.390	0.0140	0.440	0.0614	0.750	0.0776	0.990	0.0757	0.920	0.172	1.02	0.121	0.960	0.0553
Tryptophol	0.360	0.00324	0.910	0.0280	3.58	0.828	5.39	0.212	4.19	0.603	4.67	0.336	4.90	1.26
Melatonin	ND	-	ND	-	ND	-	ND	-	ND	-	ND	-	ND	-

Table S10. Concentrations of tryptophan metabolites produced by the yeast grown in YPD media with complete replacement of peptone by tryptophan and with the addition of 0.2% Tween 20 and 25 mM HEPES buffer (Run 10).

Metabolites	Concentrations (mg L^{-1})													
	0 h		4 h		8 h		12 h		16 h		20 h		24 h	
	Mean	Std	Mean	Std	Mean	Std	Mean	Std	Mean	Std	Mean	Std	Mean	Std
3-hydroxykynurenine	ND	-	ND	-	ND	-	ND	-	0.05	0.0136	0.05	0.0157	0.09	0.0741
Serotonin	ND	-	ND	-	ND	-	ND	-	ND	-	ND	-	ND	-
5-hydroxytryptophan	3.17	0.369	2.88	0.133	3.16	0.162	3.07	0.442	3.08	0.117	3.05	0.0902	3.09	0.151
Kynurenine	1.24	0.142	1.41	0.273	1.49	0.0906	1.54	0.396	1.60	0.171	1.80	0.390	2.04	0.2430
3-hydroxyanthranilic acid	0.01	0.000346	0.02	0.00552	0.03	0.00463	0.03	0.00374	0.11	0.0202	0.11	0.0600	0.31	0.276
Tryptamine	2.94	0.338	2.72	0.213	2.81	0.153	2.59	0.460	2.47	0.113	2.46	0.0788	2.58	0.1729
5-hydroxyindole acetic acid	ND	-	ND	-	ND	-	ND	-	ND	-	ND	-	ND	-
N-Acetylserotonin	ND	-	ND	-	ND	-	ND	-	ND	-	ND	-	ND	-
Xanthurenic acid	1.11	0.126	4.47	1.832	9.33	5.37	17.8	8.52	74.5	40.41	75.0	54.3	150	139
Indole-3-acetamide	ND	-	ND	-	ND	-	ND	-	ND	-	ND	-	ND	-
Kynurenic acid	0.04	0.00188	0.05	0.00251	0.06	0.0124	0.09	0.0288	0.26	0.0912	0.26	0.107	0.33	0.151
DL-Indole-3-lactic acid	3248	222	3479	1098	3397	869	2827	432	3065	331	2943	157	3123	496
Indole-3-carboxaldehyde	0.31	0.0263	0.41	0.0489	0.53	0.0563	0.53	0.0858	0.52	0.0620	0.59	0.0395	0.59	0.0418
Indole-3-acetic acid	0.32	0.0234	0.33	0.0216	0.37	0.0341	0.41	0.077	0.49	0.0610	0.50	0.00812	0.53	0.0483
Tryptophol	0.32	0.0316	0.97	0.031	2.96	0.687	4.76	1.31	8.57	0.977	7.69	0.211	8.91	1.487
Melatonin	ND	-	ND	-	ND	-	ND	-	ND	-	ND	-	ND	-

Table S11. Concentrations of tryptophan metabolites produced by the yeast grown in YPD media with complete replacement of peptone by tryptophan and with the addition of 0.2% Tween 20 and 100 mM HEPES buffer (Run 11).

Metabolites	Concentrations (mg L^{-1})													
	0 h		4 h		8 h		12 h		16 h		20 h		24 h	
	Mean	Std	Mean	Std	Mean	Std	Mean	Std	Mean	Std	Mean	Std	Mean	Std
3-hydroxykynurenine	ND	-	ND	-	ND	-	ND	-	ND	-	ND	-	ND	-
Serotonin	ND	-	ND	-	ND	-	ND	-	ND	-	ND	-	ND	-
5-hydroxytryptophan	2.65	0.0470	2.53	0.102	1.76	0.313	2.04	0.202	1.95	0.124	2.09	0.540	2.08	0.184
Kynurenine	1.37	0.0436	1.55	0.287	1.26	0.274	1.60	0.517	1.31	0.106	1.58	0.271	1.51	0.310
3-hydroxyanthranilic acid	ND	-	ND	-	0.00	-	ND	-	0.03	0.00274	0.0200	0.0198	0.110	0.130
Tryptamine	3.36	0.156	3.33	0.238	3.61	1.63	2.91	0.335	2.75	0.0900	2.90	0.529	2.64	0.283
5-hydroxyindole acetic acid	ND	-	ND	-	ND	-	ND	-	ND	-	ND	-	ND	-
N-Acetylserotonin	ND	-	ND	-	ND	-	ND	-	ND	-	ND	-	ND	-
Xanthurenic acid	ND	-	ND	-	ND	-	4.48	2.48	6.81	1.35	4.81	2.42	29.9	41.1
Indole-3-acetamide	ND	-	ND	-	ND	-	ND	-	ND	-	ND	-	ND	-
Kynurenic acid	0.02	0.00651	0.0200	0.00523	0.0200	0.00320	0.0400	0.0154	0.0400	0.00945	0.0400	0.0246	0.120	0.129
DL-Indole-3-lactic acid	3190	91.7	2719	504	2303	443	2704	678	3800	528	4207	336	3608	541
Indole-3-carboxaldehyde	0.23	0.00929	0.250	0.0336	0.290	0.0140	0.320	0.0383	0.390	0.0395	0.310	0.0185	0.280	0.0168
Indole-3-acetic acid	1.13	0.128	1.06	0.0657	0.750	0.234	0.850	0.0416	0.870	0.0288	0.970	0.250	0.820	0.112
Tryptophol	ND	-	0.0400	0.032	0.300	0.00840	0.860	0.294	1.33	0.143	1.48	0.717	2.20	0.206
Melatonin	ND	-	ND	-	ND	-	ND	-	ND	-	ND	-	ND	-

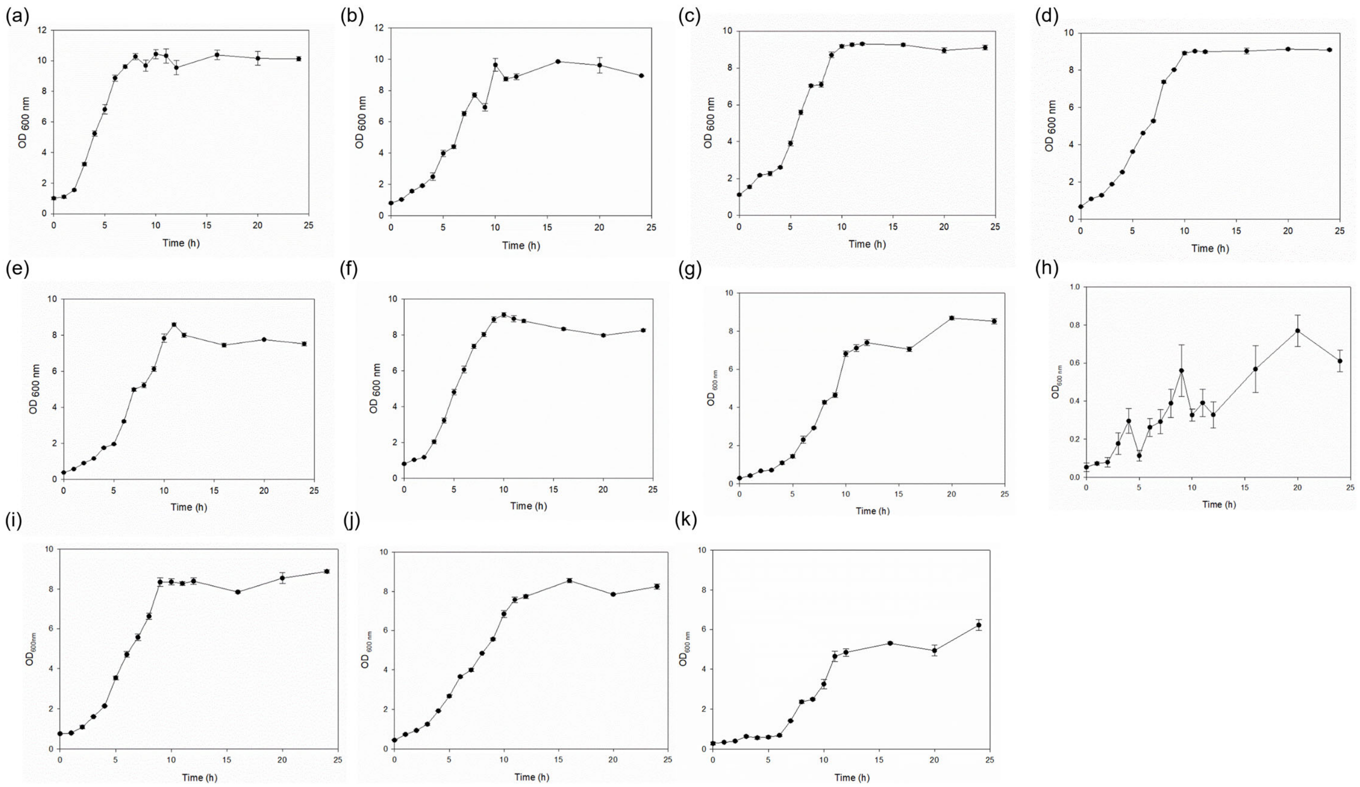

Figure S1. Growth curves of *S. cerevisiae* STG 101 under different growth conditions. The y-axis represents the optical density measured at a wavelength of 600 nm, with the x-axis denoting time in hours.

Table S12. The repeated measured ANOVA results (a) Mauchly's test of sphericity and (b) test of within-subjects effects for experimental run 3.

(a)

Mauchly's Test of Sphericity[a]

Measure: MEASURE_1

Within Subjects Effect	Mauchly's W	Approx. Chi-Square	df	Sig.	Epsilon[b]		
					Greenhouse-Geisser	Huynh-Feldt	Lower-bound
Period	.000	317.980	20	.000	.181	.185	.167

Tests the null hypothesis that the error covariance matrix of the orthonormalized transformed dependent variables is proportional to an identity matrix.

a. Design: Intercept

Within Subjects Design: Period

b. May be used to adjust the degrees of freedom for the averaged tests of significance. Corrected tests are displayed in the Tests of Within-Subjects Effects table.

(b)

Tests of Within-Subjects Effects

Measure: MEASURE_1

Source		Type III Sum of Squares	df	Mean Square	F	Sig.
Period	Sphericity Assumed	221.152	6	36.859	2.010	.072
	Greenhouse-Geisser	221.152	1.088	203.293	2.010	.175
	Huynh-Feldt	221.152	1.107	199.714	2.010	.175
	Lower-bound	221.152	1.000	221.152	2.010	.177
Error(Period)	Sphericity Assumed	1650.098	90	18.334		
	Greenhouse-Geisser	1650.098	16.318	101.123		
	Huynh-Feldt	1650.098	16.610	99.342		
	Lower-bound	1650.098	15.000	110.007		

Table S13. The repeated measured ANOVA results (a) Mauchly's test of sphericity and (b) test of within-subjects effects for experimental run 3.

(a)

Mauchly's Test of Sphericity[a]

Measure: MEASURE_1

Within Subjects Effect	Mauchly's W	Approx. Chi-Square	df	Sig.	Epsilon[b]		
					Greenhouse-Geisser	Huynh-Feldt	Lower-bound
factor1	.000	398.418	20	.000	.174	.176	.167

Tests the null hypothesis that the error covariance matrix of the orthonormalized transformed dependent variables is proportional to an identity matrix.

a. Design: Intercept

Within Subjects Design: factor1

b. May be used to adjust the degrees of freedom for the averaged tests of significance. Corrected tests are displayed in the Tests of Within-Subjects Effects table.

(b)

Tests of Within-Subjects Effects

Measure: MEASURE_1

Source		Type III Sum of Squares	df	Mean Square	F	Sig.
factor1	Sphericity Assumed	1083.127	6	180.521	1.365	.237
	Greenhouse-Geisser	1083.127	1.046	1035.227	1.365	.262
	Huynh-Feldt	1083.127	1.056	1025.327	1.365	.262
	Lower-bound	1083.127	1.000	1083.127	1.365	.261
Error(factor1)	Sphericity Assumed	11901.506	90	132.239		
	Greenhouse-Geisser	11901.506	15.694	758.345		
	Huynh-Feldt	11901.506	15.846	751.093		
	Lower-bound	11901.506	15.000	793.434		

Table S14. The mass transition ions, fragmentor voltages, and collision energies of analytes.

Analyte	Precursor ion (m/z)	Product ion (m/z)	Fragmentor (V)	C. E (V)	Internal standard
Picolinic acid	**124.1**	**78.0**	70	20	$PA-D_3$
(PA)	124.1	105.9	70	8	
3-Hydroxykynurenine	**225.2**	**208.0**	70	8	3-HKN-^{13}C2 ^{15}N
(3-HKN)	225.2	110.0	70	20	
Quinolinic acid	**168.1**	**78.0**	70	24	$PA-D_3$
(QA)	168.1	150.0	70	8	
Serotonin	**177.2**	**160.0**	60	8	Serotonin-D_4
(5-HT)	177.2	115.0	60	32	
5-Hydroxytryptophan	**221.2**	**204.0**	75	8	5-HTP-D_4
(5-HTP)	221.2	162.0	75	20	
Kynurenine	**209.2**	**192.0**	85	4	KN-D_4
(KN)	209.2	94.0	85	12	
3-Hydroxyanthranilic acid	**154.1**	**136.0**	65	8	3-HAA-D_3
(3-HAA)	154.1	80.0	65	28	
Tryptamine	**161.2**	**144.0**	65	8	TA-D_4
(TA)	161.2	115.0	65	40	
Tryptophan	**205.2**	**188.0**	75	8	Trp-D_5
(Trp)	205.2	146.0	75	16	
5-Hydroxyindole acetic acid (5-	**192.2**	**146.0**	75	16	5-HIAA-D_5
HIAA)	192.2	91.0	75	44	
Indoxyl sulfate	**211.9(**	**79.9**	-95	-24	KYNA-D_5
(IS)	211.9	132.0	-95	-20	
N-Acetylserotonin	**219.3**	**160.0**	80	12	XA-D_4
(N-AS)	219.3	115.0	80	40	
Xanthurenic acid	**206.2**	**160.0**	100	20	XA-D_4
(XA)	206.2	132.0	100	32	
Indole-3-acetamide	**175.2**	**130.0**	80	20	IAT-D_5
(IAT)	175.2	77.0	80	50	
Kynurenic acid	**190.2**	**144.0**	90	20	KYNA-D_5
(KYNA)	190.2	89.0	90	50	
DL-Indole-3-lactic acid	**205.9**	**118.0**	80	24	IAA-D_4
(ILA)	205.9	130.0	80	32	
Indole-3-carboxaldehyde (ICA)	**146.2**	**118.0**	75	16	IAT-D_5
	146.2	91.0	75	28	
Indole-3-acetic acid	**176.2**	**77.0**	75	50	IAA-D4
(IAA)	176.2	130.0	75	16	
Tryptophol	**162.2**	**144.0**	80	12	MLT-D4
(Trt)	162.2	117.0	80	28	
Melatonin	**233.3**	**174.0**	80	12	MLT-D_4
(MLT)	233.3	130.0	80	50	
5-Hydroxyindole acetic acid-D_5	**197.2**	**150.0**	70	16	
(5-HIAA-D_5)	197.2	95.0	70	44	
Serotonin-D_4	**181.2**	**164.0**	60	8	
(5-HT-D_4)	181.2	118.0	60	36	
Indole-3-acetic acid-D_4	**180.2**	**133.0**	65	16	
(IAA-D_4)	180.2	79.0	65	50	
Kynurenic acid-D_5	**195.2**	**149.0**	85	20	
(KYNA-D_5)	195.2	94.0	85	50	
Melatonin-D_4	**237.3**	**178.0**	80	16	
(MLT-D_4)	237.3	134.0	80	50	
Tryptamine-D_4	**165.2**	**148.0**	65	12	
(TA-D_4)	165.2	118.0	65	40	
Xanthurenic acid-D_4	210.2	164.0	100	20	
(XA-D_4)	210.2	136.0	100	36	
3-Hydroxyanthranilic acid-D_3 (3-	157.1	139.0	60	12	
HAA-D_3)	157.1	83.0	60	32	

**Analyte**	**Precursor ion (m/z)**	**Product ion (m/z)**	**Fragmentor (V)**	**C. E (V)**	**Internal standard**
3-Hydroxykynurenine-$^{13}C2$-^{15}N (3-HKN-$^{13}C2$ ^{15}N)	228.2	210.0	75	8	
	228.2	110.0	75	16	
5-Hydroxytryptophan-D_4 (5-HTP-D_4)	225.2	207.0	80	8	
	225.2	165.0	80	20	
Indole-3-acetamide-D_5 (IAT-D_5)	180.2	134.0	80	16	
	180.2	132.9	80	16	
Tryptophan-D_5 (Trp-D_5)	210.3	192.0	75	8	
	210.3	150.0	75	16	
